# Large-scale inference of the point mutational spectrum in human segmental duplications

**DOI:** 10.1186/1471-2164-10-43

**Published:** 2009-01-22

**Authors:** Sigve Nakken, Einar A Rødland, Torbjørn Rognes, Eivind Hovig

**Affiliations:** 1 Centre for Molecular Biology and Neuroscience, Institute of Medical Microbiology, Rikshospitalet University Hospital, NO-0027 Oslo, Norway; 2 Department of Informatics, University of Oslo, PO Box 1080 Blindern, NO-0316 Oslo, Norway; 3 Department of Tumor Biology, Institute for Cancer Research, Norwegian Radium Hospital, Montebello, 0310 Oslo, Norway; 4 Department of Medical Informatics, Norwegian Radium Hospital, Montebello, 0310 Oslo, Norway

## Abstract

**Background:**

Recent segmental duplications are relatively large (≥ 1 kb) genomic regions of high sequence identity (≥ 90%). They cover approximately 4–5% of the human genome and play important roles in gene evolution and genomic disease. The DNA sequence differences between copies of a segmental duplication represent the result of various mutational events over time, since any two duplication copies originated from the same ancestral DNA sequence. Based on this fact, we have developed a computational scheme for inference of point mutational events in human segmental duplications, which we collectively term duplication-inferred mutations (DIMs). We have characterized these nucleotide substitutions by comparing them with high-quality SNPs from dbSNP, both in terms of sequence context and frequency of substitution types.

**Results:**

Overall, DIMs show a lower ratio of transitions relative to transversions than SNPs, although this ratio approaches that of SNPs when considering DIMs within most recent duplications. Our findings indicate that DIMs and SNPs in general are caused by similar mutational mechanisms, with some deviances at the CpG dinucleotide. Furthermore, we discover a large number of reference SNPs that coincide with computationally inferred DIMs. The latter reflects how sequence variation in duplicated sequences can be misinterpreted as ordinary allelic variation.

**Conclusion:**

In summary, we show how DNA sequence analysis of segmental duplications can provide a genome-wide mutational spectrum that mirrors recent genome evolution. The inferred set of nucleotide substitutions represents a valuable complement to SNPs for the analysis of genetic variation and point mutagenesis.

## Background

Single point mutations represent a fundamental driving force for the evolution of any vertebrate genome. Mutations create DNA sequence variation that may alter gene function as well as DNA conformation and protein binding [1,2]. The spectrum of nucleotide substitutions occurring in human DNA sequences is the result of actions of various mutational sources of both endogenous and exogenous origin. An increasing body of evidence supports the idea that the majority of mutations are generated by error-prone intracellular processes that operate in a DNA sequence-dependent manner [3,4]. Examples of endogenous mutagenic processes are DNA replication (i.e. polymerase fidelity and replication slippage), post-replicative DNA mismatch repair and methylation-mediated deamination of cytosines in CpG dinucleotides [5-9]. The sequence dependence of these processes is reflected in a biased distribution of point mutations and their sequence neighbourhoods, as shown by previous analyses of pseudogene mutations, germline disease mutations and single nucleotide polymorphisms (SNPs) [3,10-13]. The nature of the observed point mutational bias is by far dominated by the hypermutability of the CpG dinucleotide [14,15]. The extent of CpG depletion in mammalian DNA attributable by methylation-mediated mutation (i.e. 5^m^C→T) is however a matter of debate [16-21]. The deficiency of CpG seen in unmethylated vertebrate DNA viruses and observations that CpG sequences are favored targets for specific exogenous mutagens suggest that other mutational and selectional mechanisms might contribute to CpG depletion [22-24]. With the exception of CpG mutations, linking the observed non-randomness of human mutations to known sequence-dependent mutational mechanisms remains challenging.

So far, large-scale genome-wide analyses of the DNA context of point mutational events have relied on either disease-causing mutations or SNP data from NCBI's dbSNP [25]. As of February 2007, dbSNP contains more than 9 million polymorphic (biallelic only) positions in the human genome. However, studies have shown that a substantial fraction of entries in dbSNP have been erroneously submitted (e.g. as a result of DNA sequencing errors), and are most likely monomorphic alleles in human populations [26,27]. Comprehensive computational analyses of SNPs may thus easily get corrupted unless a careful discrimination between validated and non-validated entries in dbSNP is undertaken.

A valuable source of information on vertebrate point mutagenesis that to our knowledge has not been thoroughly investigated is contained within human segmental duplications. Recent segmental duplications are large (≥ 1 kb) regions of high sequence identity (≥ 90%) that constitute all types of genomic elements, such as high-copy repeats and gene sequences with exon-intron structures [28-31]. Approximately 4–5% of the human genome is covered with recent duplications, being enriched in pericentromeric and subtelomeric regions of the chromosomes [32-34]. Owing to their high degree of sequence identity, a large number of mutational events can be inferred with high confidence using only pairwise DNA sequence alignments. Knowing that duplications were once identical during evolution, point mutational events correspond to mismatches in the aligned sequences. This simple approach is thus powerful for detection of a mutational spectrum in recent mammalian evolution. A proper classification of the allelic fate of newly derived alleles in segmental duplications is a different matter, however. An allele created by a point mutation in one duplication copy may be subject to a number of genetic processes that determines its allelic state in duplicated DNA. Allelic drift can take the newly derived allele through a polymorphic state (that is, SNP in a duplication) and further to fixation, in which the new allele and its counterpart in the other duplication copy are termed paralogous sequence variants (PSVs) [29,35-37]. At the same time, the newly derived allele can be distributed into multiple sequence copies by duplication or gene conversion [38-40]. The latter mechanisms take the initial mutational event into a complex type of sequence variation coined multisite variation (MSV) by Fredman and colleagues [41]. Mutational events in segmental duplications thus result in a mosaic of different genotype patterns. Altogether, data on duplication-inferred mutations generated by our approach both enriches the available pool of known mutational events within recent mammalian evolution and complements the data on disease mutations and SNPs for a contextual DNA sequence analysis of single nucleotide substitutions in humans.

We have developed a computational pipeline for inference of mutational events in segmental duplications in the human genome. The analysis of duplication-inferred mutations (DIMs) was restricted to intergenic regions of duplications, focusing on the mutational spectrum in regions that are believed to be more neutral with respect to selection forces. With the aim of detecting mutational hotspots of DIMs, we conducted a computational analysis of the local DNA sequence context of DIMs. A comparative analysis with a large set of high-quality, intergenic SNPs from dbSNP provides insights into similarities and differences between duplication-inferred variation and ordinary allelic variation in unique regions of the genome. We have also investigated the overlap between reference SNPs in segmental duplications and computationally, duplication-inferred variants. Initial reports concerning the high density of SNPs in duplications suggested that this was due to paralogous variation being misinterpreted as SNPs [28,36,37]. A following experimental study of a limited set of SNPs in duplications found that only 23% of the SNPs were consistent with paralogous variation [41], and that multisite variation appeared to be a common type of variation in these regions. We used a computational, *in silico *approach for the discovery of positional and allelic overlap between SNPs and DIMs. Our data pinpoints a large number of recorded SNPs in segmental duplications that mimic variation between paralogous sequences, and these may consequently give rise to strange patterns during traditional SNP genotyping.

## Results

### Distribution of nucleotide substitutions

**Figure 1:**
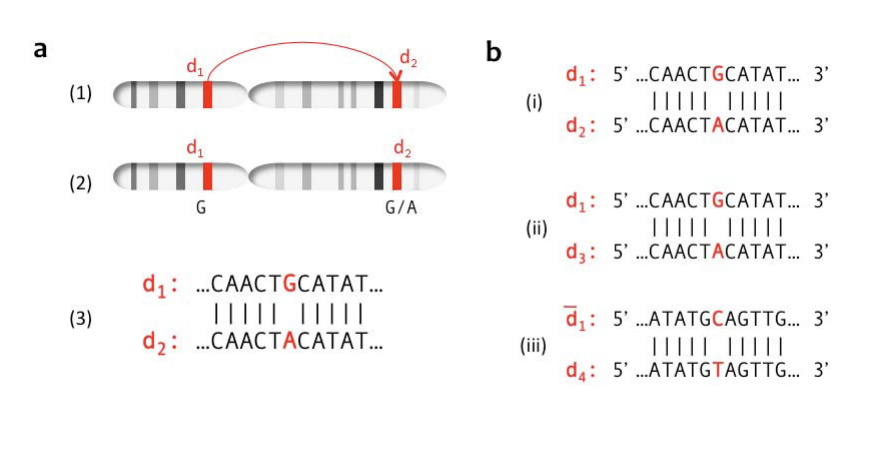
**Evolution of segmental duplications and principles for duplication-inferred mutations**. **A: **(1) An intrachromosomal duplication event occurs during evolution, followed by (2) a mutation in one of the duplication copies, causing a G/A transition. (3) A historical mutation event is inferred from an alignment of the present-day duplication copy sequences. **B: **Sequences d_1_, d_2_, d_3 _and d_4 _are all segmental duplication copies of the same ancestral DNA sequence. (i) A single base mismatch observed in the alignment between duplication copies d_1 _and d_2 _lead to the inference of a G/A (A/G) point mutational event within this DNA sequence context. (ii) An identical mismatch in the same position as observed between d_1 _and d_2 _was observed between d_1 _and d_3_. Such instances were not recorded twice in the set of mutational events, as the mismatch most likely is a propagation of the result in (i). (iii) A C/T (T/C) base mismatch in the same position as observed between d_1 _and d_2 _was observed in the alignment of d_1 _(reverse strand) and d_4_. Since the complementary mutation has been recorded in (i), we did not record this mismatch as a mutational event, as it most likely was the result of propagation by duplication.

**Figure 2:**
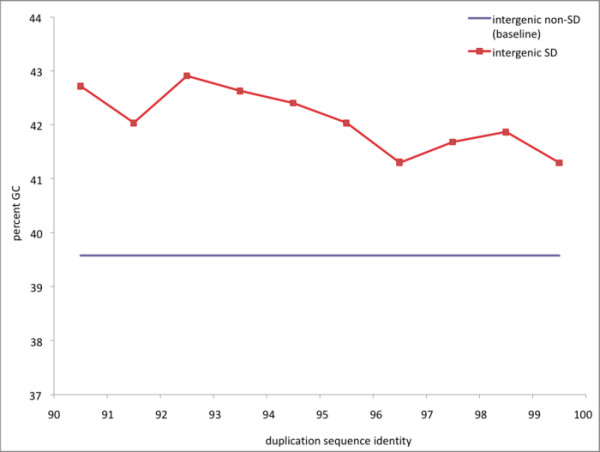
**GC content in segmental duplications compared to non-duplicated genomic regions**. GC content in intergenic regions of human segmental duplications (intergenic SD) at different levels of sequence divergence. The average GC content in non-duplicated regions is also drawn (intergenic non-SD).

**Figure 3:**
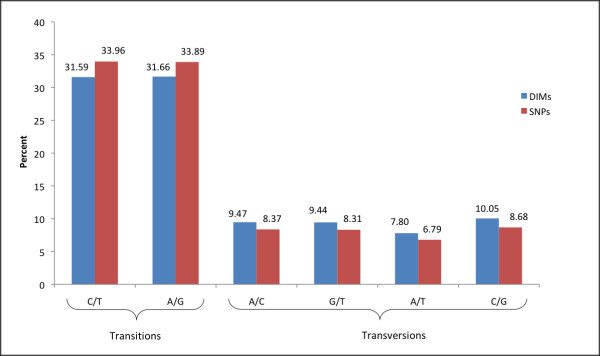
**Distribution of substitution types for DIMs and SNPs in intergenic regions**. Distribution of substitution types for 1,115,692 high-quality, genome-wide intergenic SNPs and 343,864 intergenic DIMs inferred from all human segmental duplications with sequence identity ≥ 90%. Substitution types do not carry direction (i.e. the fraction of A/G substitutions is the sum of A→G substitutions and G→A substitutions).

A total of 343,864 human duplication-inferred mutations from intergenic regions of segmental duplications satisfied the criteria we established for reliable DIM inference in DNA duplicon sequence alignments (see Figure 1 and Methods). These DIMs were subject to a comparative analysis with 1,115,692 intergenic HapMap-validated SNPs in non-duplicated regions of the human genome. The nucleotide composition of the two regions in which substitutions originated displayed a difference in GC content. Overall, intergenic regions of segmental duplications had a GC content of 41.7%, while the corresponding regions of non-duplicated DNA contained 39.6% (χ = 42,336, df = 1, p < 0.00001). When considering GC content in duplications of different levels of sequence identity, we observed a higher content at all levels (Figure 2). Figure 3 illustrates the distribution of substitution types and how the proportions of SNPs compared with inferred DIMs. Here, each type of substitution combines the nucleotide change for both directions (e.g. A/G represents the sum of all A→G and G→A substitutions) because the directions of SNPs and DIMs in our dataset are generally unknown. The histogram shows that the proportions of A/C and G/T, as well as A/G and C/T, were close to identical for SNPs. This observation reflects the complementary strand symmetry in DNA sequences as reported in previous studies on SNPs [13]. Similarly, equal proportions of complementary substitutions were observed for DIMs.

**Figure 4:**
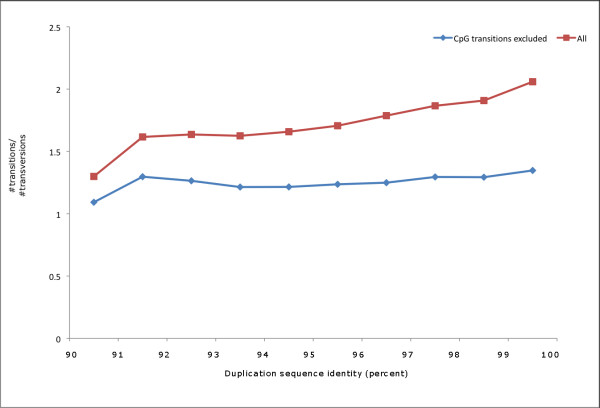
**Transitions to transversions ratio among duplication-inferred mutations**. The ratio of transitions to transversions among inferred mutations in segmental duplications at different levels of sequence divergence. The uppermost line in the plot illustrates transitions to transversions ratios when all inferred mutations were included. The lowermost line show computed ratios when transitions at the CpG dinucleotide were excluded.

As Figure 3 shows, overall DIMs and SNPs shared similar characteristics in terms of the distribution of substitution types. The two transition substitutions, A/G and C/T, account for approximately two-thirds of all substitutions for both SNPs and DIMs. Among transversions, we discovered that DIMs increased most relative to SNPs for substitutions between C and G (1.37%). The observed differences between SNPs and DIMs in terms of transition bias were noteworthy. We found that DIMs display a much smaller overall ratio of transitions over transversions than SNPs (2.11 for SNPs vs. 1.70 for all DIMs, χ = 576.7, df = 1, p < 0.00001). Estimating the transition bias with our approach ignores a potential substitution rate variation among sites, and may thus underestimate the extent of the bias. Henceforth, we emphasize the observed difference we found for SNPs and DIMs rather than the bias in itself. Furthermore, when considering the ratio of DIM transitions over DIM transversions at different levels of duplication sequence divergence, we observed a trend in which the transition bias increased when duplication divergence decreased (Figure 4). In other words, we found that DNA sequences from duplication events in most recent time had the highest degree of transition bias. To gain further insight, we established a subset of the DIMs in which transitions at the CpG dinucleotide were excluded. It is generally accepted that the CpG dinucleotide mutates at a high rate in the human genome due to deamination of 5-methylcytosine (5^m^C) to thymine, although this phenomenon has not yet been shown within the context of paralogous sequence variation. When excluding transitions at CpG dinucleotides, the trend towards increased transition bias in recent duplications was not as evident as when considering all DIMs (Figure 4).

We next determined the overall distribution of substitutions within the context of CpG dinucleotides (Table 1). Substitution frequencies were obtained in two different regions of the genome; important regulatory regions clustered with unmethylated CpGs known as CpG islands, and regions outside CpG islands. The density of CpG islands was higher in segmental duplications (1.11%) than in nonduplicated regions (0.87%, χ = 69,257, df = 1, p < 0.00001). As indicated in Table 1, the fraction of methylation-related transitions at CpG dinucleotides was much higher outside of CpG islands than within CpG islands. This was evident both in segmental duplications (DIMs) and non-duplicated regions (SNPs).

**Table 1:** Substitution frequencies at the CpG dinucleotide context

***Substitution context***	***SNPs***	***DIMs***
Non-CpG island: (A/C)G	4.69 (14,399/307,128)	5.73 (4,320/75,433)
Non-CpG island: (C/G)G	4.65 (14,270/307,128)	6.28 (4,665/75,433)
Non-CpG island: (C/T)G	**40.71 **(125,040/307,128)	**37.69 **(28,589/75,433)
Non-CpG island: C(A/G)	**40.62 **(124,746/307,128)	**37.91 **(28,713/75,433)
Non-CpG island: C(C/G)	4.58 (14,078/307,128)	4.58 (4,765/75,433)
Non-CpG island: C(G/T	4.75 (14,595/307,128)	4.75 (4,381/75,433)
CpG island: (A/C)G	9.80 (206/2,103)	9.02 (304/3,371)
CpG island: (C/G)G	13.03 (274/2,103)	14.74 (497/3,371)
CpG island: (C/T)G	**27.29 **(574/2,103)	**26.49 **(893/3,371)
CpG island: C(A/G)	**26.82 **(564/2,103)	**26.76 **(902/3,371)
CpG island: C(C/G)	13.41 (282/2,103)	14.06 (474/3,371)
CpG island: C(G/T)	9.65 (203/2,103)	8.93 (301/3,371)

Nucleotide substitution percentages at the CpG dinucleotide context are shown for intergenic DIMs and SNPs, within and outside CpG islands. The percentages of substitutions are shown along with raw counts in parentheses. Differences between islands and non-island regions for methylation-related substitutions (in boldface) are statistically significant (p < 0.00001) by Chi-square analysis.

Any observed difference between high-quality SNPs and DIMs could potentially be a result of ascertainment biases between the two mutation sets. The SNP set was ascertained using HapMap allele frequencies, excluding potential false positives originating from DNA sequencing errors. The DIM set was established using sequence alignments only, thus there is a greater chance that DIMs contain false positive mutations arising from either alignment artefacts or sequencing errors in duplications. We assessed the potential impact of noise among the duplication-inferred mutations in two different ways. First, we established two subsets of DIMs using different alignment criteria for DIM calling. The estimated, overall transition to transversion ratios in these two sets were 1.72 and 1.73. Second, we looked at three different sequence contexts that account for many false positive SNPs arising from DNA sequencing errors [27]. Having excluded all false positive SNPs in our high-quality set, we assume that the fractions of these sequence contexts in the SNP set resembles expected numbers in a human mutational spectrum. Compared with high-quality SNPs, the fraction of DIMs that occurred in these error-prone sequencing contexts increased with approximately 0.1–0.4% (Table 2).

**Table 2:** Substitution frequencies at sequence contexts associated with DNA sequencing errors

***Substitution context***	***SNPs***	***DIMs***
A(G/H)N	14.24 (158,901/1,115,692)	14.62 (50,277/343,864)
C(A/Y)C	0.38 (4,250/1,115,692)	0.45 (1,542/343,864)
G(A/C)C	1.14 (12,697/1,115,692)	1.42 (4,866/343,864)

A comparison of nucleotide substitution percentages in DIMs and high-quality SNPs at three sequence contexts previously shown to be overrepresented in false positive SNPs [27]. The percentages of substitutions are shown along with raw counts in parentheses. H stands for A, C or T, Y stands for C or T and N stands for any base.

### Sequence contexts of DIMs

We obtained DNA oligomer frequencies at SNPs and DIMs and in their corresponding reference regions to address whether both types of mutations were subject to similar mutational hotspots. Under the assumption that the middle nucleotide of an odd-length oligomer is independent of its surrounding sequence, we computed expected numbers for all oligomers. Finally, we compared the actual number for each oligomer with its expected number, defined as overrepresentation (see Methods).

**Figure 5:**
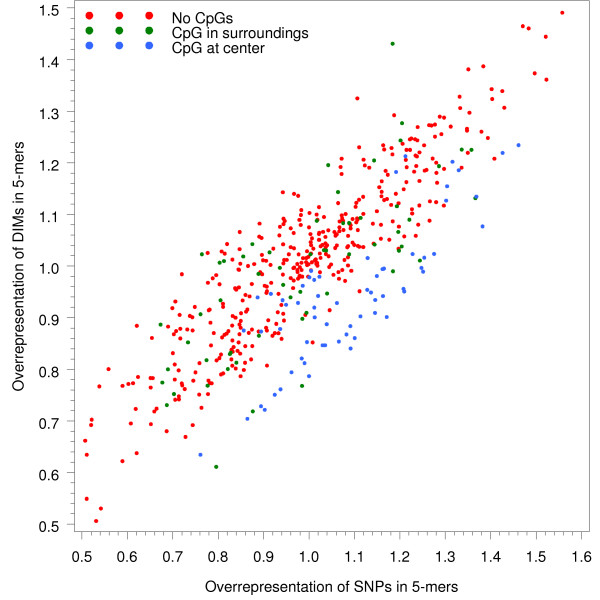
**Overrepresentation of DNA oligomers (five-mers) at sites of SNPs and recorded DIMs**. The plot compares the abundance of five-mers at substitution sites between SNPs and DIMs. Overrepresentation for a given five-mer is defined as the ratio between the number of observed five-mers with the expected number of five-mers. Five-mers are further divided into three groups; five-mers with CpG in their surroundings (e.g. CGAAT/ATTCG), those with CpG in the center (e.g. AGCGA/TCGCT) and five-mers with no CpGs.

Figure 5 compares the overrepresentation of DNA oligomers of length five (five-mers) at DIMs and SNPs. The plot illustrates similar levels of overrepresentation for the majority of five-mers at DIMs and SNPs. Oligomers where substitutions occur within the CpG dinucleotide (CpG at center) did not distribute quite as evenly between DIMs and SNPs, however. In the majority of these oligomers, SNPs were slightly overrepresented. The opposite was observed for DIMs, which occurred less frequently than expected in most of these sequences. Oligomers where substitutions take place before or after a CpG dinucleotide (CpG in surroundings) did not show any notable differences in abundance levels between SNPs and DIM.

**Figure 6:**
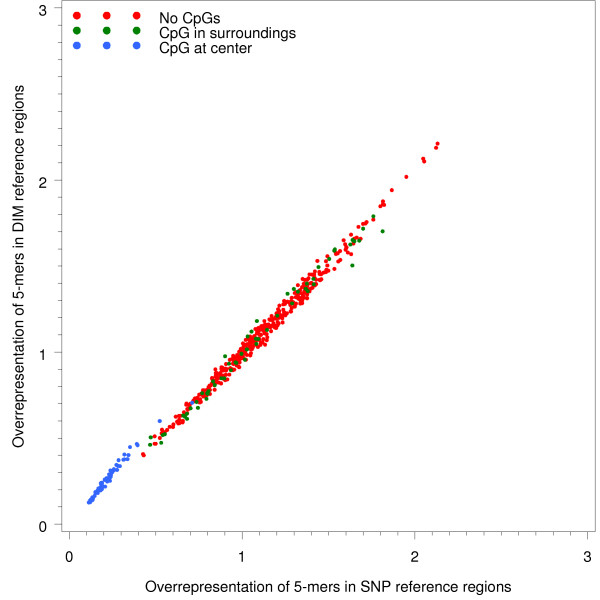
**Overrepresentation of DNA oligomers (five-mers) in duplicated and non-duplicated genomic regions**. Five-mers are divided into three groups; five-mers with CpG in their surroundings (e.g. CGAAT/ATTCG), those with CpG in the center (e.g. AGCGA/TCGCT) and five-mers with no CpGs.

We next compared the distribution of five-mers in the reference regions of SNPs and DIMs, illustrated in Figure 6. The figure shows that the set of five-mers containing no CpG, as well five-mers with CpG in surroundings were roughly equally abundant in intergenic regions of segmental duplications as in intergenic, nonduplicated regions of the genome. These five-mers were distributed close to the identity line. Five-mers affected by the CpG effect was however strongly underrepresented in both duplicated regions and non-duplicated regions. Furthermore, the degree of underrepresentation of these oligomers was slightly stronger in the regions where SNPs originate compared to the regions where DIMs originate.

### Overlap between SNPs and DIMs in segmental duplications

Previous analyses of SNPs in segmental duplications have reported an uncertainty about the validity of this particular set of SNPs [28,36,37]. The observed SNP enrichment was initially viewed as duplication-induced, representing paralogous rather than allelic variation. More advanced techniques have later shown that the spectrum of sequence variation in duplications appears as a complex combination of PSVs, SNPs in duplications and MSVs [41]. In this work, we have quantified the number of inferred mutational events in segmental duplications that overlap with reference SNPs in segmental duplications.

**Figure 7:**
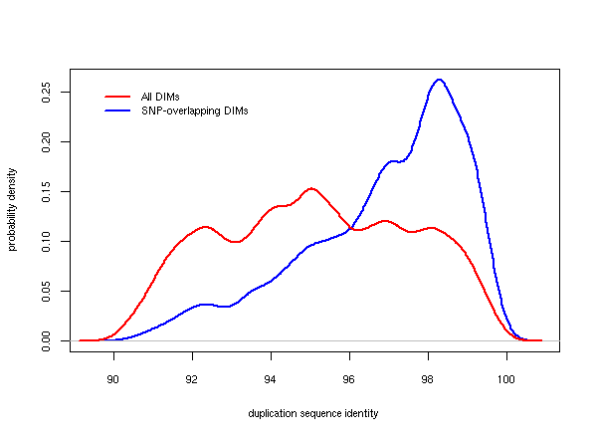
**DIMs overlap with SNPs in segmental duplications at different levels of sequence divergence**. Density distribution of the sequence divergence in which inferred mutations overlap (in terms of alleles and position) with predicted SNPs in segmental duplications. The distribution of all DIMs is shown for comparative purposes.

We retrieved a total of 458,811 SNPs from dbSNP that mapped within intergenic regions of segmental duplications. Of these SNPs, 301,968 (65.8%) were non-validated. The remaining 156,843 (34.2%) SNPs had been validated according to different criteria (see Methods). In comparison with the complementary, non-duplicated regions of the genome, which contained 31.3% nonvalidated SNPs, segmental duplications were significantly enriched for non-validated SNPs (χ = 24,952, df = 1, p < 0.00001). We then established a procedure to test whether SNPs in intergenic regions of segmental duplications coincided with DIMs. The procedure matched SNP and DIM alleles at chromosomal positions where SNPs had been identified and inferred DIMs had been recorded. Overall, we found that the chromosomal positions of 83,987 SNPs matched either a target or a source position of our inferred DIMs. Among these 83,987 SNPs, the alleles of 80,856 (96.3%) SNPs matched perfectly with corresponding DIM bases. Thus, we discovered that 17.6% of all reported intergenic SNPs in segmental duplications (80,856 SNPs out of total of 458,811) mirror sequence variation found among inferred DIMs. Although the majority of the 80,856 SNPs that overlapped with DIMs were non-validated (56,425 SNPs, 69.8%), our findings also revealed a substantial fraction of DIM overlap for validated SNPs (24,431 SNPs, 30.2%). These observations suggest that many reference SNPs in duplications most likely represent paralogous sequence variation, induced by signals from paralogous sequences in the genome. The subset of DIM-overlapping SNPs was inferred from segmental duplications that displayed a distribution dominated by duplications with 97–100% DNA sequence identity (Figure 7). All SNP entries that we found to coincide with mutational events in segmental duplications are available as supplementary material .

Fredman et al. conducted an experimental study in which they genotyped predicted SNPs in segmental duplications from fully homozygous genomes of complete hydatidiform moles (CHMs) [41]. They discovered that only 23% gave patterns indicative of PSVs, and 28% behaved differently than SNPs and PSVs, being the sum of individual genotyping signals from similar-sequence duplication copies. They termed the latter category multisite variants (MSV). Among 105 SNPs being targeted in their study, 64 SNPs mapped to the intergenic regions of duplications used in our analysis, of which they experimentally verified 11 as PSVs. We observed all 11 variants among our computationally inferred DIMs. An additional overlap was observed for 25 inferred DIMs that were designated as MSVs by Fredman and colleagues.

## Discussion

An understanding of the contextual patterns of nucleotide substitutions in the vertebrate genome is important for several reasons. The spectrum of mutational events reflect how the genome has been shaped during evolution, the mechanism of substitution mutagenesis, and it can also shed light on fundamental cellular processes such as genome stability, DNA replication and repair.

In this study, we have inferred a large set of point mutations originating within segmental duplications in the human genome. These point mutations were compared with a genome-wide collection of high-quality SNPs to assess whether these two datasets of mutational events show similar patterns in terms of distribution and surrounding sequence contexts. We initially recognized that regions of the genome covered by segmental duplications had a higher GC content than the grand average in non-duplicated regions. A previous study also reported a positive correlation between GC content and segmental duplications [34]. However, the biological interpretation of the strong association between GC content and segmental duplications is not obvious. One part may by attributed to the increased gene density in duplications [41], as regions containing genes are known to be GC-rich. Biased gene conversion may in addition play a role, a process in which repair of mismatches in heteroduplex recombination intermediates favour the fixation of G and C alleles [42,43]. Also, duplications are particularly enriched in subtelomeric regions of the chromosomes that are directly linked with GC-rich isochors [44].

The distribution of nucleotide substitutions observed in segmental duplications displays a pattern that in general is similar to SNPs. Both sets of mutations display an excess of transitional substitutions, a common phenomenon in vertebrate genomes. Among the four different transversions, the greatest difference between SNPs and DIMs were found for C/G substitutions. This finding suggests a potential association between the nucleotide composition of duplications and the frequency of substitutions, given the high GC content found in segmental duplications. Moreover, we observed a notable difference in the overall ratio of transitions to transversions between DIMs and SNPs, and an increased ratio in recently occurring DIMs. These results may reflect the evolutionary time window in which the two sets of substitutions were sampled, as well as differences in nucleotide composition between duplicated and non-duplicated DNA. Substitutions within recent segmental duplications comprise mutational events potentially originating 35–40 million years ago (≥ 90% sequence identity) up until today (100% sequence identity), and will thus include a substitution spectrum beyond the human lineage. SNPs should on the other hand represent point mutational events within the human lineage only, as they represent genetic variation between humans. If one assumes that the rate of transversions and transitions varies over time [45], one would therefore expect to see stronger long-term effects within the DIM dataset than in the SNP set. Previous studies have shown that the rate of 5^m^C deamination is limited by local GC content [46,47]. Thus, the GC richness of segmental duplications may be partly responsible for the fewer observed transitions relative to transversions.

The majority of DNA oligomers at DIM and SNP sites, respectively, displayed similar levels of abundance. This observation implies in essence that the majority of SNPs and DIMs appear to be generated by similar mutational mechanisms. We confirmed the latter in oligomers drawn from reference regions, that is intergenic regions of segmental duplications and intergenic, non-duplicated regions. However, we also discovered that many oligomers that contain substitutions at the CpG dinucleotide are overrepresented at SNPs while underrepresented at DIMs. In the reference regions, these oligomers were less underrepresented in duplications than in nonduplicated regions. As mentioned above, different effects at the CpG dinucleotide may be caused by differences in GC content, which in turn lead to different 5 mC deamination rates. Furthermore, when looking at the total mutational spectrum at the CpG dinucleotide, we observed that the frequency of methylation-related transitions differed significantly in CpG islands and non-island regions (Table 1). Our results henceforth imply that mutational events drawn from paralogous sequences exhibit the same suppression of methylation-dependent deamination in CpG islands as SNPs have been shown to do [15].

During large-scale computational identification of SNPs, many single nucleotide differences between genomic clones are taken as evidence of allelic variation and submitted to dbSNP. Without proper validation by other means, this form of SNP discovery will inevitably lead to spurious results caused by the duplication content of the human genome [26,48]. To address this issue, we systematically examined predicted SNP alleles in segmental duplications and mutations inferred from duplication alignments. Our approach revealed that nearly one out of five SNPs in duplications bear resemblance of paralogous sequence variation. Whether these SNPs behave like ordinary SNPs, MSVs or fixed PSVs is yet to be determined. Nonetheless, we suspect that traditional genotyping of the majority of these SNPs will produce misleading allele frequencies and genotype patterns since they will receive additional signals from paralogous sequences. Further, we discovered that SNPs that mirror mutational events in duplications are most prominent in duplications of high (≈97–100%) sequence identity, an observation for which we have no obvious explanation at present. In a comparative analysis with a small set of previously experimentally verified PSVs, we found all designated paralogous sequence variants among our computationally inferred mutations. In addition, we observed an overlap with computationally inferred DIMs and sites that were determined to be MSVs. The type of polymorphisms represented by MSVs involves a variation in duplication copy-number, and presumably indicates that much multisite variation may have originated from point mutational events in paralogous sequences.

Our approach does have some inherent limitations that could affect the reliability of the results obtained. These limitations involve the data source, i.e. detection of segmental duplications and reliability of DNA sequence alignments, the approach for inference of mutational events, and the sample effect. With respect to the source of segmental duplications, we relied on data provided by HGSDB [36]. The detection scheme employed by HGSDB uses BLAST for pairwise comparisons of all assembled chromosomes. Detected duplications will thus depend on the overall quality of the genome assembly, and inferred mutations will rely on correctly determined consensus sequences in the assembly. We reduced some potential assembly (and sequencing) errors by excluding high-copy repeats from the analysis, as assembly programs may fail to distinguish single base differences between repeat copies from erroneous base calls [49,50]. Since the degree of sequence divergence between duplications in HGSBB are all less than 10%, the resulting alignments are highly significant. Also, we placed restrictions on the alignment window around candidate DIMs to exclude potential alignment artefacts. Altering the alignment restrictions for DIM calling in two other DIM sets did not change the distribution of DIM substitutions to a large extent. In error-prone DNA sequencing contexts we observed a small increase of DIMs relative to high-quality SNPs, suggesting a minor impact of random noise in the DIM set. Altogether, we believe that the sequence alignments did not cause any serious errors.

Computational inference of mutational events leading to DIMs also has limitations. First of all, the directionality of the mutations was not inferred with our approach, i.e. an A→T mutation could not be distinguished from a T→A mutation. Thus, an observed (C/T)G substitution may not necessarily reflect the deamination of a methylated thymine, but rather correspond to a thymine to cytosine transition. A recent study of the directionality of SNPs indicated that most substitutions in intergenic regions have roughly the same amounts of substitutions in either direction [11]. Whether DIMs display the same characteristics is unknown. Secondly, when the same mutational events were found propagated in several duplications (Figure 1B), we excluded them as individual events under the assumption of no multiple substitutions at a single site. This assumption is not likely to be violated in DNA sequences that show as low degree of sequence divergence as recent segmental duplications.

The sample of inferred DIMs were, as mentioned above, retrieved from all human chromosomes in regions where duplications have been found to exist. The total number of DIMs sampled was so large (≈344,000) that we believe they can provide a general pattern of substitutions in segmental duplications. In contrast to unique DNA sequences, duplicated sequences frequently undergo homology-driven mutation when involved in either non-allelic homologous recombination or gene conversion [28,42]. In the latter process, DNA repair of nucleotide mismatches in heteroduplex DNA intermediates has been shown to be GC-biased, providing a direct link to the GC-richness of duplications [51]. Investigating the relationship between biased repair and the observed distribution of DIMs requires further work, considering that base mispairs are corrected with different efficiencies and specificities in mammals [52]. The inferred point mutational spectrum was restricted to intergenic regions, excluding all DIMs located within RefSeq transcripts. Among all DIMs inferred, we thus omitted nearly 31.5% in our analyzed sample, as they all originated within UTRs, exons and introns residing in segmental duplications. As shown in early studies of molecular evolution, regions under functional constraints (i.e. human transcripts) show different patterns and rates of substitutions from selectively neutral sequences such as pseudogenes [53,54]. In order to establish a neutral pattern of point mutations in segmental duplications, minimized with the confounding effects of natural selection, we excluded any mutational event in which either of the nucleotides were found inside RefSeq transcripts. Since the point mutational spectrum in coding regions of segmental duplications may display different characteristics than what we found in intergenic regions, we suggest that these nucleotide substitutions should be explored in further work.

Most important, our computational analysis of segmental duplications in the human genome suggests that they can be utilized as a novel data source for the analysis of vertebrate point mutagenesis. There are essentially two different observations that support this claim. First, the distribution and context of computationally inferred DIMs and a set of high-quality set of SNPs in intergenic regions of the genome were largely similar (Figures 3, 5 and 6). Second, we found that a large fraction of the inferred DIMs overlap with verified SNPs, which provides evidence that our inference strategy is able to retrieve actual mutational events that lead to genetic variation. Moreover, our inferred set of nucleotide substitutions originates from regions in all human chromosomes, as segmental duplications are not restricted to any particular chromosome, but rather distributed in a genome-wide fashion. We believe that the inferred dataset of point mutations may be a valuable complement to SNPs for the analysis of human genetic variation.

## Methods

### Segmental duplication data

The Human Genome Segmental Duplication Database (HGSDB, ) has been reported to contain chromosomal coordinates of all segmental duplications (length ≥ 5 kb and sequence identity ≥ 90%) in the human genome, based on a computational detection scheme [36]. In total, 12589 unique pairwise sequence alignments of duplication copies were downloaded from HGSDB (build hg17). The two sequences in any pairwise sequence alignment of duplications were denoted as source and target sequences. 6587 alignments had both source and target sequences located on the same chromosome (intrachromosomal duplications), the remaining 6002 alignments had their duplication copies on nonhomologous chromosomes (interchromosomal duplications). Several regions were involved in both inter- and intrachromosomal duplications. The average alignment length was approximately 20.5 kb. The total nonredundant content of recent segmental duplications was found to be 133.9 Mb, comprising 4.7% of the non-gap length (2851.3 Mb) of the human genome. Chromosomal coordinates of RefSeq transcripts and CpG islands annotated to hg17 were downloaded as flat files from the UCSC genome browser  and mapped to segmental duplications from HGSDB. High-copy repeats in segmental duplications were identified as lower-case nucleotides (output from RepeatMasker) within alignments downloaded from HGSDB.

### Inference of mutational events in segmental duplications

Mutational events were inferred using DNA sequence alignments from HGSDB only. Figure 1A illustrates the basic inference principle. Since no other mammalian genome was used in our analysis, we did not attempt to infer the directionality of the mutational events or separate events that originated within different vertebrate lineages. We merely inferred that mutational events had occurred since the duplication event took place. Two other factors related to the nature of segmental duplications had further impact on how DIMs were recorded (see Figure 1B). We wrote software for the traversal of pairwise DNA sequence alignments and recording of all mutational events along with their neighbouring sequence context (total entries n = 800,649). The dataset was reduced by excluding DIMs occurring in RefSeq transcripts as well as high-copy repeats as masked by RepeatMasker (n = 548,088). An alignment window of length 40 around each candidate DIM was extracted. To ensure that inferred DIMs were results of actual point mutational events rather than alignment artefacts, we only kept DIMs where the 40 bp alignment window satisfied the following criteria: (1) maximum four mismatches, (2) maximum two gaps (indels) and (3) no mismatches in the three immediate positions upstream and downstream of the candidate DIM site. With these criteria, the total number of intergenic inferred DIMs was 343,864. To test whether these alignment criteria induced any bias in the distribution of DIM types, we established two control sets in which DIMs were inferred in a stricter manner. In the first control set, we required a minimum of seven non-variant bases upstream and downstream of the candidate DIM site (258,612 DIMs), and in the second control set we increased this number to fifteen (108,117 DIMs).

To ensure that substitutions were sampled consistently across alignments with different sequence identity, we calculated the overall transition to transversion ratio for DIMs as a weighted sum of ten different bin ratios. DIMs were initially put in ten bins according to the sequence alignment identity in which they originated (i.e. 90 to 100), and a ratio for each bin was calculated without weighting. Each bin was then assigned a weight, representing the expected fraction of all substitutions that originated from alignments in the given bin. The expected number of substitutions in an alignment was estimated as alignment length multiplied by the fraction of nonidentical bases (the expected number in a bin was found by summing over all bin alignments).

### SNP data

The human dbSNP database (build 126) was downloaded as XML files and parsed with Perl scripts for retrieval of biallelic RefSNP entries (reference SNPs). We established two different sets of SNP data. The first set contained a high-quality set of SNPs in non-duplicated regions of the genome, used for a comparative sequence context analysis with DIMs. The second set contained all reference SNPs in segmental duplications.

In the high-quality set of SNPs, we decided to only keep entries that were validated within the HapMap project [55]. We excluded all ambiguously mapped SNPs, that is, polymorphic sites where the flanking sequences did not map to a unique region in the genome with an alignment identity of at least 99% (total entries n = 2,160,150). The fraction of SNPs where allele frequencies in none of the four HapMap populations satisfied the basic SNP definition, that is, minor allele frequency ≥ 1%, were also omitted (as these may not mirror true SNP sites). The number of SNPs was further reduced by excluding SNPs that mapped within RefSeq transcripts (n = 1,337,235), SNPs where the flanking sequence (100 bp) fell inside high-copy repeats as masked by RepeatMasker (n = 1,131,893), and finally SNPs inside segmental duplications (n = 1,115,692).

A second set of SNPs was established by fetching all reference SNPs located within intergenic regions of segmental duplications, both validated and nonvalidated (n = 458,811). A SNP was classified as nonvalidated within dbSNP if it did not satisfy any of the following criteria: 1) allele frequencies in a given population, 2) multiple independent submissions, or 3) both alleles seen in at least two chromosomes. An overlap between a SNP and a DIM was considered valid if the chromosomal position of the SNP matched either the source or the target position associated with the DIM, and that the alleles at the SNP and DIM site matched (either directly or in a complementary manner if the SNP and DIM were recorded on different strands).

### Sequence context of nucleotide substitutions

We determined whether similar mutational mechanisms act upon segmental duplications as in non-duplicated genomic regions by quantifying the frequencies of DNA oligomers at DIMs and high-quality SNPs. For comparison, we counted reference oligomer frequencies in the surrounding regions of DIMs (intergenic, duplicated DNA) and SNPs (intergenic, non-duplicated DNA).

Let *uxv *represent a *k*-mer where *x *is the middle nucleotide and *u *and *v *are surrounding nucleotides, and *u*[*xy*]*v *represent a *k*-mer where the middle nucleotide is a substitution pair *x*/*y*. Let *n*(*uxv*) count the number of *k*-mers that are either *uxv *or its reverse complement, and define *n*(*u*[*xy*]*v*) similarly. We count SNPs and DIMs separately.

The nucleotide and substitution pair probabilities are *p*(*x*) = *n*(*x*)/*n *and *p*([*xy*]) = *n*([*xy*])/*n *for reference region and substitution respectively, with *n *the corresponding total number of nucleotides. Note that by this definition f.ex. *p*(*A*) = *p*(*T*) is the probability of any nucleotide being either *A *or *T*, each with a 1/2 probability of being on either strand. If the middle nucleotide, *x *or [*xy*], is independent of the surrounding nucleotides, the expected numbers in the reference regions are

*μ*(*uxv*) = *n*(u*v)·*p*(*x*)/2

where *n*(u*v) is the sum of all *n*(*uxv*) for different *x*, and the division by two is because there is a 1/2 chance that *x *is on the same strand as u*v. For substitutions,

*μ*(*u*[*xy*]*v*) = *n*(*u*[**]*v*)·*p*(*xy*)/2

except that if either [*xy*] or u*v are their own reverse complements one should not divide by 2. The overrepresentation (or abundance) is defined as *R*(*uxv*) = *n*(*uxv*)/*μ*(*uxv*), and *R*(*u*[x*]*v*) = *n*(*u*[x*]*v*)/*μ*(*u*[x*]*v*) where *u*[x*]*v *indicates the sum over all matching *u*[*xy*]*v *for *n *and *μ*.

## Authors' contributions

EH conceived the study and outlined data analysis tasks. SN performed data retrieval and statistical analysis with help from EAR and TR. EH and TR provided feedback on results obtained. SN and EH drafted the manuscript together. All authors read and approved the final manuscript.
